# Prevalence and predictors of headache in patients referred to polysomnography

**DOI:** 10.1186/1129-2377-14-90

**Published:** 2013-11-18

**Authors:** Kornelia Katalin Beiske, Michael Bjørn Russell, Knut Stavem

**Affiliations:** 1 Department of Neurology, Medical Division, Akershus University Hospital, Lørenskog, Norway; 2 Head and Neck Research Group, Research Center, Akershus University Hospital, Lørenskog, Norway; 3 Institute of Clinical Medicine, Campus Akershus University Hospital, University of Oslo, Nordbyhagen, Norway; 4 Department of Pulmonary Medicine, Akershus University Hospital, Lørenskog, Norway; 5 Health Services Research Unit, Akershus University Hospital, Lørenskog, Norway

**Keywords:** Obstructive sleep apnea, Polysomnography, Headache, Headache frequency

## Abstract

**Background:**

The objectives of this study were; (1) to assess the prevalence and frequency of headache in patients referred to polysomnography (PSG) due to a clinical suspicion of obstructive sleep apnea (OSA) or another sleep disturbance and compare with a reference population, and (2) to assess the association of OSA severity with headache and headache frequency.

**Methods:**

A total of 784 participants filled in a headache questionnaire between 2003 and 2009 at the Department of Clinical Neurophysiology, Akershus University Hospital. Of these patients 477 were suspected to have OSA, and 307 had other sleep complaints. We assessed the prevalence of headache and monthly headache frequencies, as well as sleep apnea severity using an apnea-hypopnea index (AHI). The association of headache and monthly headache frequencies with PSG subgroups was assessed using multivariate logistic and ordered logistic regression analysis.

**Results:**

The frequency of headache was not associated with the severity of OSA. Patients referred to a sleep study for any reason had higher odds ratio (OR) for having experienced headache during the past year than population controls after adjustment for age, gender and education, i.e. patients with normal AHI had OR of 3.56, patients with OSA had OR of 3.51, and patients with other sleep disturbances had OR of 3.33. Similarly, the adjusted OR of being in a higher category of monthly headache frequency compared to controls was higher in those with normal AHI (OR 3.42), OSA (OR 3.29), and other sleep disturbances (OR 3.00).

**Conclusions:**

The odds of headache and headache frequency were higher in subjects referred to a PSG for any sleep disturbance independently of OSA, compared to general population controls. However, there was no association between experiencing headache during the past year or headache frequency with OSA severity.

## Background

Obstructive sleep apnea (OSA) is characterized by forceful snoring and excessive daytime sleepiness with an increased propensity to fall asleep during the daytime. OSA is defined as repeated episodes of obstructive apnea and/or hypopnea during sleep, leading to sleep disruption [1]. It is categorized using the apnea-hypopnoea index (AHI), which is calculated from the results of an overnight polysomnography (PSG). Cut-off values for AHI varies, but usually AHI <5 is normal, while mild, moderate and severe OSA is defined as AHI 5–14, AHI 15-29 and AHI ≥ 30 [2,3]. The prevalence of OSA is 4-10% in middle-aged Caucasians from general populations, and it increases with age [4-8].

It is still controversial whether patients with OSA experience more headache than people from the general population. Several studies show increased headache frequency in patients who snore, as well as in patients with OSA, compared to general population samples [9-12], while other studies have not been able to reproduce these findings [13,14]. Chronic headache is associated with the presence or absence of PSG-confirmed OSA, independent of apnea severity and oxygen desaturation [15]. The mean nocturnal values of SpO₂ are significantly lower in patients with than without morning headache [16], suggesting an association with OSA severity. Other recent population-based studies report that morning headaches are more frequent among patients with than without OSA [17], but the severity of OSA is not associated with the frequency of tension-type headache or migraine [17-19]. Although many studies concentrate on OSA and its association with a variety of headache types, headache frequency is also increased in other sleep disorders such as periodic limb movement disorder, circadian rhythm disorder, insomnia, and hypersomnia [20]. This motivated us to examine the presence of headache and headache frequency in a population of patients referred to a sleep study due to any sleep disorder.

The study objectives were to assess; (1) the prevalence and frequency of headache in patients referred to PSG due to a clinical suspicion of OSA or another sleep disturbance and compare with a reference population, and (2) the association of OSA severity with headache and headache frequency.

## Methods

### Participants

#### Polysomnography population

**Figure 1:**
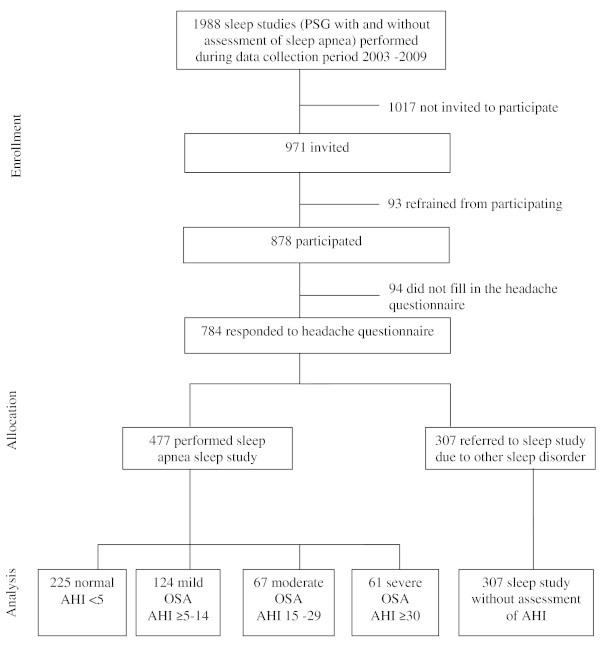
**Flow chart of the PSG population.** PSG = polysomnography, OSA = obstructive sleep apnea, AHI = apnea-hypopnea index.

Figure 1 shows a flow chart of the study. The present study aimed to include all subjects referred to a PSG for any sleep disturbance between 2003 and 2009. A total of 1988 sleep studies were performed at the Department of Clinical Neurophysiology, Akershus University Hospital during this period. The patients were referred mainly from general practitioners, neurologists, or ear-nose-throat specialists. Sleep complaints other than OSA investigated by PSG at the Department of Clinical Neurophysiology include restless legs syndrome, insomnia, bruxism, RBD (rapid eye movement behavior disorder) and circadian rhythm sleep disorders. Unfortunately neither the diagnosis nor the reason for referral was noted during the data collection process.

A total of 878 patients consented to participate, 93 patients refused to participate, and 1017 were not invited. In total, 784 participants filled in the headache questionnaire; 477 were suspected to have OSA, and 307 had other sleep complaints.

The inclusion process was delegated to technicians at the Department of Clinical Neurophysiology. Examination of inclusion data showed that a substantial decrease in inclusion occurred every summer holiday in July and August. The inclusion rates were highest between 2003 and 2007. Only 49% of the patients who had PSG during the total inclusion period, were asked to participate, and 90% of them agreed to participate in the study. It is unknown whether all of the patients registered as non-invited were truly not invited, or if they simply did not fill in the short questionnaire described below.

#### Control group

A control group of 287 persons, aged 24 to 87 years, 63% men and 37% women, from the general population in the hospital’s catchment area, was drawn from the Norwegian National Population Register in 1997 and used for comparison in the present study. This control group originally served as controls in a study of patients following head trauma [21]. The PSG population patients and general population controls filled in the same self-reported headache questionnaire.

### Questionnaire

Patients in the PSG population responded to a questionnaire including items about demographics, smoking, use of continuous positive airway pressure (CPAP) equipment as well as the validated Norwegian version of the Epworth Sleepiness Scale (ESS) [22]. The headache questionnaire included a question about having experienced headache during the past year, and if affirmative was followed by a question about average headache frequency per month during the past year with the following response options: <1 day, 1-7 days, 8-14 days, more than 14 days per month, or every day/nearly every day.

The first page of the full questionnaire was also requested filled in by patients who refrained from participation. This part of the questionnaire included information about their age, gender, height, weight, complaints leading to a PSG referral (snoring, feeling tired during the daytime, falling asleep during the daytime, falling asleep while driving, concentration problems, cessation of breathing during the night, accident/near accident while driving, other sleep complaints) and whether they suffer from chronic illness.

Patients in the PSG population, who were only evaluated for sleep apnea, underwent an ambulant *sleep apnea recording* using the Embla system (EMBLA; Medcare-Flaga; Reykjavik, Iceland), recording airflow with thermistors, oxygen saturation by finger-pulse oximeter, heart rate, abdominal and thoracic respiratory movements, body position and snoring.

Patients with symptoms suspect for both OSA and other sleep disorders were referred to a *combined sleep apnea and polysomnography* (PSG), while patients not suspect for OSA were referred to a *simplified PSG without sleep apnea recording* using the ambulant Embla system. These recordings included surface electrode recordings of the electroencephalogram (C3, C4, O1, O2), electro-oculograms (EOG1 and EOG2), electrocardiogram, as well as *m. submentalis* and bilateral *m. tibialis anterior* electromyograms. All patients completed sleep and medication logs for 2 weeks prior to the sleep apnea/PSG recordings.

These PSG recordings were scored according to the criteria of the American Academy of Sleep Medicine Task Force by physicians specializing in clinical neurophysiology [23].

### Headache classification

The headache was classified to either of the following main groups [21,24,25]:

(1) no headache

(2) infrequent headache (headache <1 day per month)

(3) frequent headache (1-14 days per month)

a. 1-7 days per month

b. 8-14 days per month

(4) chronic headache (>14 days per month)

### Statistical analysis

Descriptive statistics are presented using percent (number) or mean (SD). For headache frequencies, we present 95% confidence interval for the frequencies in percent for groups according to AHI, using Wilson confidence intervals [26]. To assess the association between the PSG population subgroups and headache, we assessed the odds of experiencing headache (headache during the past year vs. none) in populations categorized according to AHI strata–normal (AHI < 5), OSA (AHI ≥5) and unknown (patients without AHI assessment, due to suspicion of sleep disorders other than OSA)–compared with the general population controls (referent) using multivariate logistic regression analysis. We adjusted for age, gender and education that based on the literature were possible confounders [27-32].

Among subjects that reported headache frequency (patients n = 645, controls n = 183), we assessed the association between PSG population subgroups and categories of headache frequency using multivariate ordered logistic regression analysis with categorized monthly headache frequency, (<1 day, 1-7 days, 8-14 days, > 14 days) as the dependent variable. As independent variables, we used populations categorized according to AHI strata–normal (AHI < 5), OSA (AHI ≥5) and unknown (patients without AHI assessment, due to suspicion of sleep disorders other than OSA)–compared with the general population controls (referent). Again, we adjusted for age, gender and education in the analysis (see above). The proportional odds assumption was assessed using the Brant test.

The ordered logistic analysis is interpreted as the odds of being in groups greater than *k* versus those who are in groups less than or equal to *k*, where *k* is the level of the response variable. This is constant across all levels of the dependent variable. For example, in our analysis the proportional odds ratio represents the odds of having headache >7 days per month vs. ≤ 7 days per month (and also the odds of having headache >14 days vs. ≤ 14 days per month).

Among patients undergoing a sleep study due to suspected OSA, we assessed the association between OSA severity and experiencing headache during the past year using multivariate logistic regression analysis, adjusting for age, sex and education. Finally, we assessed the association between OSA severity and categorized monthly headache frequency (<1 day, 1-7 days, 8-14 days, >14 days) among those undergoing a sleep study due to suspected OSA using multivariate ordered logistic regression analysis, as described above, also adjusting for age, sex and education.

We chose a 5% significance level using two-sided tests, and used Stata version 12 (College Station, TX) for all analyses.

## Results

Descriptive data of the two PSG populations are shown in Table 1. Patients with suspected OSA (n = 477) were on average older (p < 0.0001), comprised a higher proportion males (p < 0.0001) and were more likely to snore (p < 0.0001) than patients without suspected OSA (n = 292). There was no difference in the level of education (p = 0.19) or smoking status (p = 0.46) between these two groups.

**Table 1:** Descriptive statistics for patiens who answered questions about headache frequency and were referred for sleep study due to a clinical suspicion of obstructive sleep apnea (N = 477) or other sleep disorder (N = 292)

		**Obstructive sleep apnea**			**Other sleep study**
	**Normal**	**Mild**	**Moderate**	**Severe**	**Unknown**
AHI	<5	5-14	15-29	≥30	
n	225	124	67	61	292
Age, years [mean (SD)]					
Male	42.9 (12.2)	49.1 (11.2)	52.0 (10.1)	51.1 (11.0)	40.7 (12.7)
Female	43.2 (12.6)	51.1 (10.7)	56.0 (8.4)	50.9 (7.9)	41.2 (12.4)
Body mass index, kg/m^2^, mean (SD) (n = 761)	27.3 (5.0)	28.4 (4.6)	29.9 (4.6)	32.7 (5.7)	25.8 (5.2)
Male gender (n = 472)	56 (126)	70 (87)	84 (56)	92 (56)	50 (147)
Education (n = 760)					
Schooling up to 10 years	39 (87)	46 (57)	46 (30)	53 (31)	42 (123)
High school degree	17 (37)	15 (19)	11 (7)	15 (9)	20 (59)
University or college education	44 (97)	39 (48)	44 (29)	32 (19)	37 (108)
Employment (n = 757)					
Student	5 (11)	2 (2)	0 (0)	2 (1)	4 (32)
Homemaker	3 (6)	1 (1)	2 (1)	0 (0)	3 (8)
Retired/sick leave/disablement benefits	26 (57)	33 (41)	28 (18)	31 (18)	34 (97)
Unemployed	1 (2)	3 (4)	3 (2)	0 (0)	5 (13)
Employed part time	12 (26)	12 (15)	12 (8)	3 (2)	10 (30)
Employed full time	53 (117)	49 (61)	55 (36)	64 (38)	43 (124)
Current smoker (n = 253)	34 (76)	30 (37)	25 (17)	37 (22)	35 (101)
Epworth Sleepiness Scale >10 (n = 378)	51 (114)	47 (58)	48 (32)	59 (36)	47 (138)

Percent (number), unless otherwise stated.

There was no difference (p = 0.94) in age between the 877 participating patients and those 1017 not invited to participate, with mean (SD) age of 55.1 (12.9) and 45.1 (16.7), respectively (p = 0.94). There was also a similar allocation of gender among participants and those not invited, 61% and 57% males respectively (p = 0.07).

Patients referred to PSG for evaluation of OSA had slightly higher average ESS scores compared to those with other sleep other disorders (PSG without assessment of AHI) (p = 0.07). These patients also had higher mean body mass index (BMI) than those with other sleep disorders (p < 0.0001). Increasing BMI was correlated with increasing AHI.

The prevalence of no, infrequent, frequent and chronic headache in the mild, moderate and severe AHI groups showed only minor differences with overlapping confidence intervals (Table 2).

**Table 2:** Headache frequency in patients with suspected obstructive sleep apnea (N = 477), percent

		**Obstructive sleep apnea**		
	**Normal**	**Mild**	**Moderate**	**Severe**
AHI	<5	5-14	15-29	≥30
n	225	124	67	61
	% (95% CI)	% (95% CI)	% (95% CI)	% (95% CI)
No headache	13 (9-18)	17 (11-25)	19 (12-30)	23 (14-35)
Infrequent headache				
<1 day per month	20 (16-26)	18 (12-25)	31 (22-43)	20 (12-31)
Frequent headache				
1-7 days per month	36 (30-42)	41 (33-50)	24 (15-35)	26 (17-38)
8-14 days per month	12 (8-16)	11 (6-17)	9 (4-18)	10 (5-20)
Chronic headache				
>14 days per month	19 (15-25)	14 (9-21)	16 (9-27)	21 (13-33)

CI denotes confidence intervals.

Patients in the PSG population referred to a sleep study for any reason had higher odds of suffering from headache during the past year than general population controls (Table 3). Patients with normal AHI, mild, moderate or severe OSA had similar odds ratio (OR) for having experienced headache during the past year compared to controls, after adjustment for age, gender and education. Similarly, the OR of being in a higher category of headache frequency, categorized according to monthly headache frequencies, was 3.42 (p <0.001) for patients with normal AHI, 3.29 (p < 0.001) for patients with OSA, and 3.00 (p < 0.001) for patients with other sleep disorders compared to controls, after adjustment for age, gender and education (Table 4). The OR for headache was higher in women than men, and higher in patients with < 12 years than ≥12 years of education.

**Table 3:** Odds of experiencing headache during the past year in patients referred to a sleep study compared to general population controls

	**n**	**Odds ratio**	**95% confidence interval**	**p**
Population				
General population controls	287	1		
Patients: normal sleep study	222	3.56	(2.20-5.76)	<0.001
Patients: obstructive sleep apnea	253	3.51	(2.29-5.38)	<0.001
Patients: sleep study without assessment of AHI	300	3.33	(2.14-5.20)	<0.001
Age, increase of 1 year	1062	0.96	(0.95-0.97)	<0.001
Gender				
Male	652	1		
Female	410	2.72	(1.87-3.97)	<0.001
Education				
0-9 years	474	1		
10-12 years	184	1.06	(0.64-1.74)	0.825
>12 years	404	0.69	(0.49-0.98)	0.040

Multivariate logistic regression analysis, N = 1062.

**Table 4:** Odds of being in a higher category of monthly headache frequency, categorized as < 1 day, 1-7 days, 8-14 days, >14 days, among those reporting headache

	**n**	**Odds ratio**	**95% confidence interval**	**p**
Population				
General population controls	183	1		
Patients: normal sleep study	191	3.42	(2.33-5.04)	<0.001
Patients: obstructive sleep apnea	201	3.29	(2.21-4.88)	<0.001
Patients: sleep study without assessment of AHI	254	3.00	(2.09-4.31)	<0.001
Age, increase of 1 year	829	1.00	(0.99-1.01)	0.65
Gender				
Male	474	1		
Female	355	1.59	(1.22-2.07)	0.001
Education				
0-9 years	373	1		
10-12 years	153	0.60	(0.42-0.85)	0.005
>12 years	303	0.62	(0.47-0.83)	0.001

Multivariate ordered logistic regression analysis, N = 829^*^.

*Fewer patients compared to Table 3 due to omission of answers to headache frequency questions.

Analysis of men and women separately showed the same pattern as described above with significantly increased OR for headache in patients referred to a sleep investigation compared to general population controls. This was valid for both men and women, when analysing headache during the past year as the main outcome, as well as headache frequency (data not shown).

The adjusted OR of having experienced headache during the past year among patients with mild, moderate or severe OSA was not statistically significant different compared to patients with normal AHI (Table 5). Also the adjusted ORs of being in a higher category of headache frequency categorized according to monthly headache frequency (<1 day, 1-7 days, 8-14 days, >14 days), were similar among patients with mild, moderate or severe obstructive sleep apnea compared to patients with normal AHI (Table 6).

**Table 5:** Odds of experiencing headache during the past year among patients with suspected OSA

	**n**	**Odds ratio**	**95% confidence interval**	**p**
Normal (AHI <5)	222	1		
Mild OSA (AHI 5-14)	126	1.13	(0.59-2.17)	0.71
Moderate OSA (AHI 15-29)	66	1.26	(0.58-2.76)	0.56
Severe OSA (AHI ≥ 30)	61	1.00	(0.46-2.18)	0.99

OSA = obstructive sleep apnea.

Multivariate logistic regression analysis, adjusted for age, sex, and education (N = 475).

**Table 6:** Odds of being in a higher category of monthly headache frequency, categorized as < 1 day, 1-7 days, 8-14 days, >14 days, among patients with suspected OSA and headache

	**n**	**Odds ratio**	**95% confidence interval**	**p**
Normal (AHI <5)	192	1		
Mild OSA (AHI 5-14)	104	1.01	(0.65-1.58)	0.96
Moderate OSA (AHI 15-29)	53	0.86	(0.46-1.61)	0.63
Severe OSA (AHI ≥ 30)	46	1.19	(0.63-2.23)	0.59

OSA = obstructive sleep apnea.

Multivariate ordered logistic regression analysis, adjusted for age, sex, and education (N = 395).

## Discussion

We found increased odds of headache and headache frequency in patients referred to sleep investigation regardless of the sleep disturbance, compared to controls from the general population and adjusted for age, gender and education. An association between headache/headache frequency and severity of OSA could not be demonstrated.

Our findings are consistent with previous findings of increased prevalence of chronic headache in patients referred to PSG with and without PSG-confirmed OSA [15]. The lack of association between AHI scores and headache is also in accordance with previous case–control studies [15,17,33]. The finding of higher odds of headache in women than men, and lower odds with increasing highest attained education, are also in accordance with previous studies [30,32]. Studies that have not shown an association between OSA and headache have been community studies of patients reporting symptoms of OSA, or patients primarily referred to a neurologist due to headache complaints who were later diagnosed with OSA [13,14].

The reason for increased headache prevalence among patients referred to PSG for any sleep disturbance compared to general population controls is still unclear. According to a recent paper sleep disorders can amplify nociception and worsen sleep disorders [34]. This suggests that poor sleep quality due to any causative factor may contribute to increased prevalence and frequency of headaches. Another reason may be Berkson’s bias, which describes an ascertainment or sampling bias, meaning that patients with sleep disorders and complaints of headache may more likely to be referred to a sleep study than patients with sleep disorders alone [35].

The headache questionnaire used in this study provided information about experiencing headache during the past year as well as headache frequency, but not about headache type. This headache questionnaire was nearly identical to the headache questionnaire used in an epidemiological study in Norway, and found to be both reliable and valid [24]. Previous studies have used questionnaires that gave more detailed information about the clinical characteristics of headaches associated with sleep apnea [15,17].

Invitation for participation in this study was by chance, and not subject to selection for particular sleep complaints or sleep disorders. The relatively high number of patients who were not asked to participate for reasons unknown is a potential limitation of the study, although analysis of age and gender of patients not invited were similar to participants. Patients not invited were referred to the same types of sleep studies as participating patients. The strength of this study is the inclusion of a relatively large sample of patients referred to PSG, who filled in the same headache questionnaire used by general population controls.

A recent review suggests that chronic headaches can be regarded as soft signs of a sleep disorder [36]. Our findings in this patient population referred to a sleep study due to any sleep disorder are in agreement with this statement. The presence of frequent or chronic headache may therefore give reason for physicians to question sleep disturbance as a possible contributor to headache complaints.

## Conclusions

The odds of headache and headache frequency were higher in patients referred to a PSG for any sleep disturbance, including patients with and without PSG-confirmed OSA, compared to a control sample from the general population. However, there was no association between experiencing headache during the past year or headache frequency with sleep apnea severity as defined using the AHI, among patients with PSG-confirmed OSA.

## Competing interests

The authors declare that they have no competing interests.

## Authors’ contributions

KKB and KS initiated the study. KKB contributed to data collection, analysis and organization. KKB also drafted the manuscript. KS performed the majority of the statistical analysis and revised the manuscript. MBR provided expert advice and critically revised the manuscript. All authors read and approved the final manuscript.
